# Second-order work in barodesy

**DOI:** 10.1007/s11440-018-0749-z

**Published:** 2018-12-18

**Authors:** Gertraud Medicus, Barbara Schneider-Muntau, Dimitrios Kolymbas

**Affiliations:** Division of Geotechnical and Tunnel Engineering, University of Innsbruck, Technikerstr. 13, 6020 Innsbruck, Austria

**Keywords:** Barodesy, Constitutive model, Finite element simulations, Second-order work

## Abstract

Second-order work analyses, based on elasto-plastic models, have been frequently carried out leading to the result that failure may occur *before* the limit yield condition is encountered. In this article, second-order work investigations are carried out with barodesy regarding standard element tests and finite element applications. In barodesy, it was shown—like in hypoplasticity and elasto-plasticity—that second-order work may vanish at stress states inside the critical limit surface. For boundary value problems, an end-to-end shear band of vanishing second-order work marks situations, where failure is imminent.

## Introduction

Investigations, based on elasto-plastic models, have been carried out by several authors [2–4, 10, 17, 25, 35] leading to the result that failure may occur *before* the limit yield condition is encountered. Second-order work investigations with hypoplasticity showed similar results [2, 7, 14, 28], among other things that for loose soil second-order work vanishes at stress states inside the critical limit surface.

The literature on uniqueness, stability, bifurcation and failure is vast. Stability refers to systems, as characterized by their boundary conditions, and not only to materials. In particular, the nature of tractions on the boundary plays an important role, e.g. the question whether they are dead or follower loads or not. In this article, we consider the second-order work expressed as ${\hbox {tr}}({\mathring{{\mathbf {T}}}}{\mathbf {D}})$. For symbols and notation, see Sect. 2. Many relevant citations can be found in Hill [9]. As they refer mainly to elastic solids, we cite some publications from soil mechanics.

Lade [15], based on experimental results, concludes that the violation of the stability criteria of Hill, ${\hbox {tr}}({\mathring{{\mathbf {T}}}}{\mathbf {D}})\ge 0$, and Drucker, ${\hbox {tr}}({\mathring{{\mathbf {T}}}}{\mathbf {D}}^{{\mathrm{pl}}})\ge 0$, does not necessarily evoke an observable collapse of the sample.

Nova [29] investigated the controllability of element tests of soils obeying the constitutive law ${\mathbf {D}}={\varvec{\mathcal {C}}}{\mathring{{\mathbf {T}}}}$, with ${\varvec{\mathcal {C}}}$ being the fourth-order compliance matrix. As *loading program*, he denotes the prescription of *n* components of ${\mathbf {D}}$ (or linear combinations thereof) and the remaining $6-n$ components of ${\mathring{{\mathbf {T}}}}$ (or linear combinations thereof), with $0\le n \le 6$. The response to this loading program consists of the corresponding $6-n$ components of ${\mathring{{\mathbf {D}}}}$ and the remaining *n* components of ${\mathring{{\mathbf {T}}}}$. He concludes that the constitutive relation determines a *unique* response, if the symmetric matrix ${\varvec{\mathcal {C}}}^{{\rm s}}$ is positive definite:

1 $$\begin{aligned} {\mathbf {D}}\, {\varvec{\mathcal {C}}}\, {\mathbf {D}}> 0 \end{aligned}$$

or, equivalently, if the ‘second-order work’ $W_2$ is positive:

2 $$\begin{aligned}&W_2={\hbox {tr}}({\mathring{{\mathbf {T}}}}{\mathbf {D}})\nonumber \\&\quad ={\mathring{T}}_{11}D_{11}+{\mathring{T}}_{22}D_{22}+{\mathring{T}}_{33}D_{33}+ 2{\mathring{T}}_{12}D_{12}+2{\mathring{T}}_{23}D_{23}+2{\mathring{T}}_{13}D_{13} \nonumber \\&\quad >0. \end{aligned}$$

The unique invertibility (in the sense of *unique* response to *any* loading program) was called by Nova ‘controllability’. This term is, however, somehow misleading, as it can be easily conceived as the condition for obtaining a unique deformation of a sample by application of boundary tractions and displacements (in the sense that the deformation of a body is controlled by the boundary tractions and displacements). Such deformation is in many cases expected to be homogeneous, the corresponding tests are then called ‘element tests’. The unique solution of a boundary value problem with prescribed boundary displacements does, however, not follow from $W_2>0$ but from another condition, firstly derived by Hill [9], as shown here in a simplified form: we consider the velocity field ${\mathbf {v}}$ as the solution of a boundary value problem and investigate whether this solution is unique. Assume that there exists also another solution ${\bar{{\mathbf {v}}}}\ne {\mathbf {v}}$. Denoting differences by a prime, e.g. ${\mathbf {v}}'={\mathbf {v}}-{\bar{{\mathbf {v}}}}$, we observe that ${\mathbf {v}}'$ vanishes at the boundary. The equilibrium equation reads $\nabla \cdot {\mathbf {T}}=\mathbf{0}$, and continued equilibrium reads $\nabla \cdot {\dot{{\mathbf {T}}}}=\mathbf{0}$. The same equations hold also for the stress difference ${\mathbf {T}}':={\mathbf {T}}-{\bar{{\mathbf {T}}}}$: $\nabla \cdot {\mathbf {T}}'=\mathbf{0}$ and $\nabla \cdot {\dot{{\mathbf {T}}}}'=\mathbf{0}$. We now consider the integral $I:=\int _V \nabla \cdot ({\dot{{\mathbf {T}}}}'{\mathbf {v}}'){\text {d}}V$ and apply the theorem of Gauss. We thus obtain that this integral vanishes:

3 $$\begin{aligned} \int _V \nabla \cdot ({\dot{{\mathbf {T}}}}'{\mathbf {v}}') \ {\text {d}}V=\int _S {\mathbf {T}}'{\mathbf {v}}' \cdot {\mathbf {n}}\ {\text {d}}S =0, \end{aligned}$$

because ${\mathbf {v}}'=\mathbf{0}$ on the surface *S*. Further,

4 $$\begin{aligned} I = \int _V \nabla \cdot ({\dot{{\mathbf {T}}}}'{\mathbf {v}}') \ {\text {d}}V = \int _V {\dot{{\mathbf {T}}}}'\cdot \nabla {\mathbf {v}}' \ {\text {d}}V + \int _V {\mathbf {v}}' \ \nabla \cdot {\dot{{\mathbf {T}}}}'\ \ {\text {d}}V = 0 \end{aligned}$$

The second integral on the right-hand side vanishes due to continued equilibrium. Thus, for non-uniqueness must hold:

5 $$\begin{aligned} \int _V {\dot{{\mathbf {T}}}}'\cdot \nabla {\mathbf {v}}' \ {\text {d}}V\equiv \int _V {\dot{{\mathbf {T}}}}'\cdot {\mathbf {D}}' \ {\text {d}}V = 0, \end{aligned}$$

which is impossible if ${\dot{{\mathbf {T}}}}'\cdot {\mathbf {D}}'>0$ holds everywhere [the notation ${\dot{{\mathbf {T}}}}'\cdot {\mathbf {D}}'$ denotes the same as tr$({\dot{{\mathbf {T}}}}'{\mathbf {D}}')$]. Hence the condition $C_1$

6 $$\begin{aligned} {\hbox {tr}}({\dot{{\mathbf {T}}}}'{\mathbf {D}}')>0 \end{aligned}$$

implies uniqueness. For the special case ${\bar{{\mathbf {v}}}}=\mathbf{0}$ we have: tr$({\dot{{\mathbf {T}}}}'{\mathbf {D}}')=$ tr$({\dot{{\mathbf {T}}}}{\mathbf {D}})$. Hence,

7 $$\begin{aligned} {\hbox {tr}}({\dot{{\mathbf {T}}}}'{\mathbf {D}}')>0\,\leadsto\,{\hbox {tr}}({\dot{{\mathbf {T}}}}{\mathbf {D}})>0 \end{aligned}$$

Thus we have

8 $$\begin{aligned} C_1\,\leadsto\,\hbox {uniqueness} \end{aligned}$$

and

9 $$\begin{aligned} C_1\,\leadsto\,{{\hbox {tr}}}({\dot{{\mathbf {T}}}}{\mathbf {D}})>0. \end{aligned}$$

However, the statement that ${{\hbox {tr}}}({\dot{{\mathbf {T}}}}{\mathbf {D}})>0$ implies uniqueness cannot be generally inferred, e.g. uniqueness is implied if the material is incrementally linear.

Negative second-order work denotes softening, i.e. the tangential stiffness has at least one negative eigenvalue. For some static boundary conditions of dead loads, this implies increasing in kinetic energy and thus collapse. In fact, Nicot et al. [26] correlate vanishing second-order work with increase of kinetic energy and corresponding failure.

## Symbols and notation

We use the symbolic notation for Cauchy effective stress ${\mathbf {T}}$ and stretching ${\mathbf {D}}$. In the figures, the more familiar symbol $\sigma _i$ instead of $T_i$ is used for the principal stresses. Normal stresses are defined negative for compression. Tensors are written in bold capital letters (e.g. ${\mathbf {X}}$). $|{\mathbf {X}}|:=\sqrt{{\hbox {tr}}{\mathbf {X}}^2}$ is the Euclidean norm of ${\mathbf {X}}$, ${\hbox {tr}}{\mathbf {X}}$ is the sum of the diagonal components of ${\mathbf {X}}$. The superscript 0 marks a normalized tensor, i.e. ${\mathbf {X}}^0 = {\mathbf {X}}/|{\mathbf {X}}|$. ${\mathbf {1}}$ denotes the second-order unit tensor. Stresses are considered as effective ones, the normally used dash is omitted. The stretching tensor ${\mathbf {D}}$ is the symmetric part of the velocity gradient. Stretching ${\mathbf {D}}$ is only approximately equivalent to the strain rate ${\dot{\varvec{\varepsilon }}}$. For rectilinear extensions however, ${\mathbf {D}}$ equals ${\dot{\varvec{\varepsilon }}}$, considering the logarithmic strain $\varvec{\varepsilon }$. $p:=-\frac{1}{3}{\hbox {tr}}{\mathbf {T}}$ is the mean effective stress, $\varepsilon _{\text {vol}}={\hbox {tr}}\varvec{\varepsilon }$ is the volumetric strain. For compressive strain, $\varepsilon _i$ is defined negative.

The deviatoric stress is written as $q=-(\sigma _1-\sigma _3)$ and the deviatoric strain reads $\varepsilon _q=-2/3\cdot (\varepsilon _1-\varepsilon _3)$ for axisymmetric conditions.

Note that several definitions for dilatancy can be found. In barodesy is used $\delta :={\hbox {tr}}{\mathbf {D}}^0={{\dot{\varepsilon }}_{\text {vol}}}/{|{\dot{\varvec{\varepsilon }}}}|$. A common definition of the angle of dilatancy $\psi$ for axisymmetric triaxial compression, i.e. ${\dot{\varepsilon }}_1<0$, is, see e.g. [23]:

10 $$\begin{aligned} \dfrac{{\dot{\varepsilon }}_{\text {vol}}}{{\dot{\varepsilon }}_{\text {q}}}=\dfrac{-6\sin \psi }{3-\sin \psi }. \end{aligned}$$

This ratio of volumetric to deviatoric strain rate can be expressed in dependence of $\delta$, (for $\delta >0$) as:

11 $$\begin{aligned} \dfrac{{\dot{\varepsilon }}_{\text {vol}}}{{\dot{\varepsilon }}_{\text {q}}} = \frac{-3\delta }{\sqrt{6-2\delta ^2}}. \end{aligned}$$

From Eqs. 10 and 11 follows:

12 $$\begin{aligned} \delta =\frac{2\sqrt{2}\sin \psi }{\sqrt{3-2\sin \psi +3\sin ^2\psi }}. \end{aligned}$$

## Barodesy

Similar to hypoplasticity, barodesy^1^ is a constitutive relation of the form ${\mathring{{\mathbf {T}}}}={\mathbf {h}}({\mathbf {T}}, {\mathbf {D}}, e)$, which is based on the asymptotic behaviour of soil [12, 13, 20]. The general form of the barodetic constitutive relation is:

13 $$\begin{aligned} {\mathring{{\mathbf {T}}}}= h\cdot (f{\mathbf {R}}^0+g{\mathbf {T}}^0)\cdot |{\mathbf {D}}| \end{aligned}$$

${{\mathbf {R}}}^0$ and ${\mathbf {T}}^0$ are the normalized tensors of proportional stress paths ${{\mathbf {R}}}$ and stress ${\mathbf {T}}$, respectively. The tensor ${{\mathbf {R}}}$ depends on ${\mathbf {D}}$, and the relation ${{\mathbf {R}}}({\mathbf {D}})=-\exp (\alpha \,{\mathbf {D}}^0)$ includes a stress–dilatancy relation [21], with $\alpha$ as a scalar quantity. The scalar quantities *h*, *f* and *g* depend on the actual stress level, the actual void ratio *e* and a stress-dependent critical void ratio $e_{\mathrm{c}}$ and, thus describe barotropy and pyknotropy.^2^ Appendix 1 summarizes all equations of barodesy. Regarding second-order work, we have to check whether the expression

14 $$\begin{aligned} {\hbox {tr}}({\mathring{{\mathbf {T}}}{\mathbf {D}}})= h\cdot \left( f\cdot {\hbox {tr}}({\mathbf {R}}^0{\mathbf {D}})+g\cdot {\hbox {tr}}({\mathbf {T}}^0{\mathbf {D}})\right) \cdot |{\mathbf {D}}| \end{aligned}$$

can become negative or equal to zero. In barodesy, the second-order work is zero when *Y* (Eq. 15) is zero:

15 $$\begin{aligned} Y=\frac{{\hbox {tr}}({\mathring{{\mathbf {T}}}{\mathbf {D}}})}{h\cdot g \cdot \vert {\mathbf {D}}\vert ^2}:= \frac{f}{g}{\hbox {tr}}({\mathbf {R}}^0{\mathbf {D}}^0)+{\hbox {tr}}({\mathbf {T}}^0{\mathbf {D}}^0)=0. \end{aligned}$$

The variables *f*, *g* and ${{\mathbf {R}}}^0$ depend on ${\mathbf {D}}^0$. We therefore may rewrite equation 15 as:

16 $$\begin{aligned} k({\mathbf {D}}^0)+{\hbox {tr}}({\mathbf {T}}^0{\mathbf {D}}^0)= 0. \end{aligned}$$

To determine the boundary of the region with $W_2>0$ in stress space, we consider a stress state ${\mathbf {T}}$ and ask whether there is a ${\mathbf {D}}$ such that Eq. (16) be *just* fulfilled. ‘Just fulfilled’ means that there is *only one solution*${\hat{{\mathbf {D}}}}$. This implies

17 $$\begin{aligned}&k({\hat{{\mathbf {D}}}}^{0})+{\hbox {tr}}({\mathbf {T}}^{0}{\hat{{\mathbf {D}}}}^{0})=0 \quad \hbox {and} \quad k({\mathbf {D}}^{0})+{\hbox {tr}}({\mathbf {T}}^0{\mathbf {D}}^0)<0 \nonumber \\&\quad {\hbox {for}} \quad {\mathbf {D}}^0\ne {\hat{{\mathbf {D}}}}^{0}. \end{aligned}$$

Equation (16) introduces a relation between ${\mathbf {D}}^0$ and ${\mathbf {T}}^0$, that is not necessarily unique. However, the additional requirement (17) imposes uniqueness and, thus, establishes a function

18 $$\begin{aligned} {\hat{{\mathbf {D}}}}^0={\mathbf {h}}({\mathbf {T}}^0). \end{aligned}$$

As in Eq. 18${\mathbf {T}}^0$ is a function of ${\hat{{\mathbf {D}}}}^0$, it can be written as

19 $$\begin{aligned} {\mathbf {T}}^0=a\cdot {\mathbf {1}}+b\cdot {\hat{{\mathbf {D}}}}^{0}+c\cdot {\hat{{\mathbf {D}}^0}^2}, \end{aligned}$$

according to the theorem of Cayley–Hamilton. The scalars *a*, *b* and *c* depend on the invariants of ${\hat{{\mathbf {D}}}}^0$. Equation 19 is only valid for vanishing second-order work. It therefore follows for $W_2=0$, that (for isotropic materials) ${\mathbf {T}}^0$ and ${\hat{{\mathbf {D}}}}^0$ are coaxial.

As in Sect. 4 only rectilinear extensions are examined, the co-rotational, objective stress rate $\mathring{{\mathbf {T}}}$ coincides with $\dot{{\mathbf {T}}}$. This follows from ${\mathring{{\mathbf {T}}}}={\dot{{\mathbf {T}}}}+{\mathbf {T}}{\mathbf {W}}-{\mathbf {W}}{\mathbf {T}}$ and ${\mathbf {W}}={\mathbf {0}}$. In general cases (e.g. the finite element applications in Sect. 5), we do consider the Zaremba–Jaumann rate ${\mathring{{\mathbf {T}}}}$.

Several rectilinear deformations represented by the principal stresses $T_1$, $T_2$, $T_3$ will be numerically examined as to whether there can be found ${\mathbf {D}}^0$-tensors such that tr$({\dot{{\mathbf {T}}}}{\mathbf {D}}^0)= 0$. The boundary of the region in stress space with tr$({\dot{{\mathbf {T}}}}{\mathbf {D}}^0)> 0$ is the surface of vanishing second-order work.

## Element tests

We investigate the second-order work for specific loading paths in standard element tests (Sects. 4.1–4.2) and give a more general perspective in Sect. 4.3.

### Undrained triaxial test

The undrained triaxial test is an illustrative example to explain vanishing second-order work inside the critical stress surface. As it is a rectilinear extension, we set ${\mathbf {D}}=\dot{\varvec{\varepsilon }}$. For an undrained test applies ${\hbox {tr}}{\mathbf {D}}=0$ (i.e. ${\dot{\varepsilon }}_{\text {vol}}={\dot{\varepsilon }}_1+2 {\dot{\varepsilon }}_2=0$) and therefore ${\dot{\varepsilon }}_2=-1/2\cdot {\dot{\varepsilon }}_1$.

20 $$\begin{aligned} {\hbox {tr}}({\dot{{\mathbf {T}}}}{\mathbf {D}})={\dot{T}}_{1}\cdot {\dot{\varepsilon }}_1+2\cdot {\dot{T}}_{2}\cdot {\dot{\varepsilon }}_2={\dot{T}}_{1}\cdot {\dot{\varepsilon }}_1-{\dot{T}}_{2}\cdot {\dot{\varepsilon }}_1= -{\dot{q}}\cdot {\dot{\varepsilon }}_1. \end{aligned}$$

**Fig. 1:**
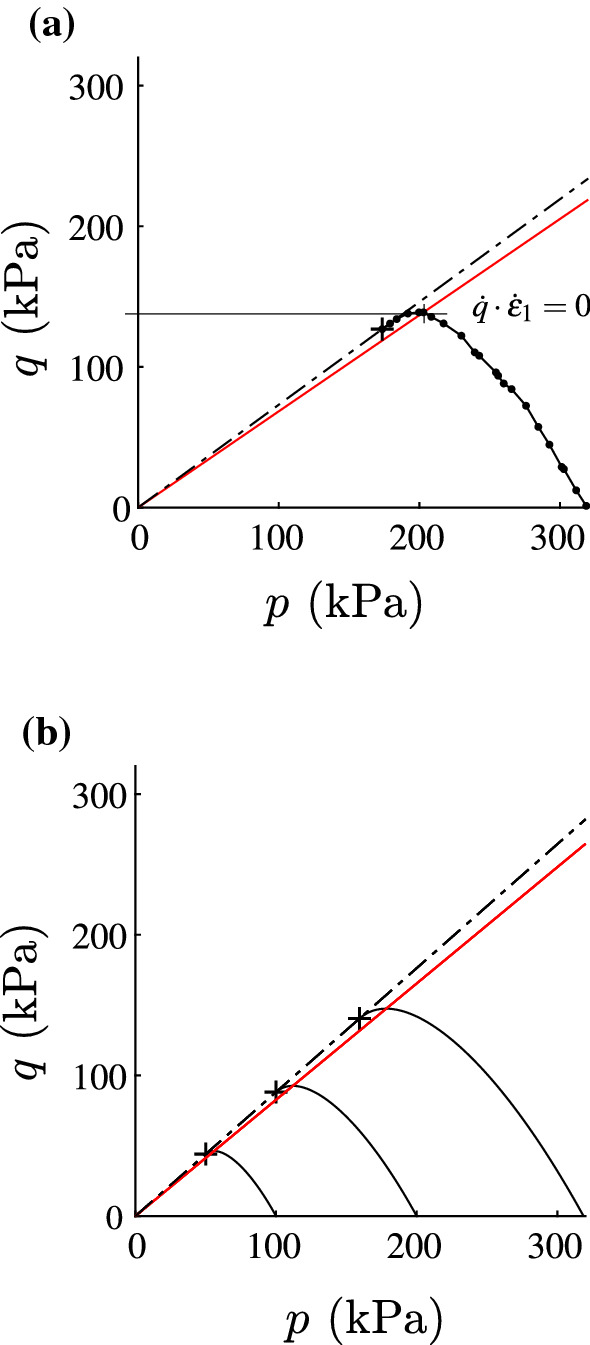
Normally consolidated CU tests: at the maximum of *q* (${\dot{q}} =0$ and ${\dot{\varepsilon }}_1\ne 0$), ${\hbox {tr}}({\dot{{\mathbf {T}}}}{\mathbf {D}})$ vanishes. The crosses $+$ mark the critical stress states. The solid line is the locus of vanishing second-order work, the dot-dashed line is the critical state line, i.e. $q=M\cdot p$ with $M=6\sin \varphi _{\mathrm{c}}/(3-\sin \varphi _{\mathrm{c}})$ for triaxial axisymmetric compression. The experimental results in **a** refer to London clay by Gasparre [6] (test *r10nc*), in **b** normally consolidated London clay is simulated with barodesy [20]

From Eq. 20 follows ${\hbox {tr}}({\dot{{\mathbf {T}}}}{\mathbf {D}})=0$ for ${\dot{q}} =0$, cf. Fig. 1. Tests controlled with dead loads are not possible beyond the maximum of *q* (${\dot{q}} =0$ and ${\dot{\varepsilon }}_1\ne 0$). The experimental results in Fig. 1a refer to London clay by Gasparre [6], in Fig. 1b normally consolidated London clay samples are simulated with barodesy [20], cf. Table 1 for parameters. The solid line marks the points of vanishing second-order work, the dot-dashed line is the critical state line. The maximum of *q* (${\dot{q}}=0$ and ${\hbox {tr}}({\dot{{\mathbf {T}}}}{\mathbf {D}})=0$) is clearly visible. Note that the mobilized friction angle at the maximum of *q* is lower than at critical state (marked with the crosses $+$ in Fig. 1).

**Table 1:** Critical state soil mechanics parameters used for the calibration of *barodesy*

Material	$\varphi _{\text {c}}$	*N*	$\lambda ^*$	$\kappa ^*$	Source
London clay	22.6$^\circ$	1.375	0.11	0.016	Mašín [18]
Weald clay	24$^\circ$	0.8	0.059	0.018	Mašín [19]

In Appendix 2, we add the drained triaxial test as an illustrative example to investigate second-order work in barodesy.

### Non-conventional drained triaxial tests

**Fig. 2:**
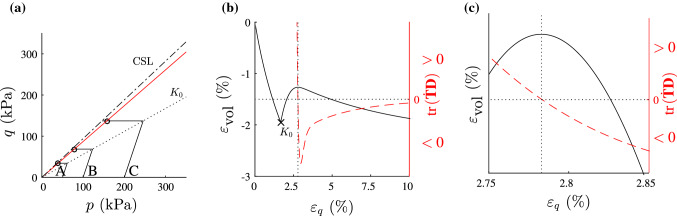
Simulations of non-conventional drained triaxial tests of Weald clay ($\varphi _{\mathrm{c}}=24^\circ$, see Table 1 for parameters) with barodesy: all samples are initially normally consolidated with 50 kPa, 100 kPa and 200 kPa. In **a** stress paths of tests A, B and C are displayed and stress states with ${\hbox {tr}}({\dot{{\mathbf {T}}}}{\mathbf {D}})=0$ are marked with circle, in **b** the volumetric behaviour (solid line) and ${\hbox {tr}}({\dot{{\mathbf {T}}}}{\mathbf {D}})$ (broken line) of test A are shown. The $K_0$ state is marked in **a** and **b**. **c** Detail of **b** where it is visible that $W_2=0$ at a local maximum of ${\dot{\varepsilon }}_{\text {vol}}$

We consider drained triaxial tests with reduction of *p* at $q=$ const. For normally consolidated Weald clay (for parameters see Table 1) we set $p_{\text {ini}}=50$ kPa, $p_{\text {ini}}=100$ kPa and $p_{\text {ini}}=200$ kPa. The tests start as conventional drained triaxial tests and at $\sigma _1=1/K_0\cdot \sigma _2$ the mean effective stress *p* is decreased by increasing the pore pressure, cf. similar experiments by Lade [15] and simulations by Wan and Pinheiro [34]. A reduction in the mean stress is obtained e.g. in the case of an excavation [8]. For the tests in Fig. 2a, the deviatoric stress remains constant ($q=75$ kPa for test A, $q=150$ kPa for test B, $q=300$ kPa for test C), hence ${\dot{q}}=0$. The second-order work according to equation 37 simplifies thus to ${\hbox {tr}}({\dot{{\mathbf {T}}}}{\mathbf {D}})={\dot{p}} \cdot {\dot{\varepsilon }}_{\text {vol}}$. With decreasing *p*, i.e. ${\dot{p}} \ne 0$, the second-order work vanishes for this specific loading path at ${\dot{\varepsilon }}_{\text {vol}}=0$. Simulations with barodesy show that in the non-conventional drained triaxial tests of Fig. 2, the second-order work vanishes inside the critical limit surface.

Figure 2a shows the stress paths of the non-conventional triaxial tests. The stress states with ${\hbox {tr}}({\dot{{\mathbf {T}}}}{\mathbf {D}})=0$ are marked with circles ($\circ$) and connected with a line. In Fig. 2b, c the solid line shows the volumetric strain and the dashed line the second-order work of test A. It is visible that second-order work is zero at the local maximum of $\varepsilon _{\text {vol}}$, i.e. ${\dot{\varepsilon }}_{\text {vol}}=0$, which has experimentally been confirmed: a sudden collapse is reported to occur [3, 17] at the local maximum of volumetric strain. The mobilized friction angle at ${\hbox {tr}}({\dot{{\mathbf {T}}}}{\mathbf {D}}) =0$ is smaller than the mobilized friction angle at critical state, cf. Fig. 2a.

### Investigations in the deviatoric plane

The following analysis has been carried out numerically. We consider the deviatoric plane ${\hbox {tr}}{\mathbf {T}}=-500$ kPa $=$ const. in the principal stress space spanned by $T_1, T_2, T_3$ and we search for the boundary of the region where tr$({\dot{{\mathbf {T}}}}{\mathbf {D}})>0$. We examine stress rays starting from the hydrostatic axis $T_1=T_2=T_3$. On each ray, we step forward with small increments of deviatoric stress. At each step, we check whether there are ${\mathbf {D}}$ tensors such that the condition tr$({\dot{{\mathbf {T}}}}{\mathbf {D}})=0$ is fulfilled.

To this end, it is sufficient to check tensors ${\mathbf {D}}^0$, coaxial to ${\mathbf {T}}$, distributed in all directions of the space $D_1,D_2,D_3$, with the magnitude $\vert {\mathbf {D}}\vert =1$, the polar angle $0<\theta <\pi$, and azimuth angle $0<\phi <2\pi$. The principal values of ${\mathbf {D}}$ are:

21 $$\begin{aligned} D_1= \sin \theta \cdot \cos \phi \end{aligned}$$

22 $$\begin{aligned} D_2= \sin \theta \cdot \sin \phi \end{aligned}$$

23 $$\begin{aligned} D_3= \cos \theta \end{aligned}$$

We vary $\theta$ and $\phi$ independently on each stress ray for every step and search for minimum values of second-order work. As soon as tr$({\dot{{\mathbf {T}}}}{\mathbf {D}})=0$ is encountered, the stress state ${\mathbf {T}}$ belongs to the searched boundary.

The Mohr–Coulomb yield locus in the deviatoric plane corresponds to a hexagon and the mobilized friction angle $\varphi _{\mathrm{m}}$ reads^3^:

24 $$\begin{aligned} \sin \varphi _{\mathrm{m}}=\frac{T_{\text {min}}-T_{\text {max}}}{T_{\text {min}}+T_{\text {max}}}. \end{aligned}$$

In barodesy, the cone of critical stress states practically coincides with the locus according to Matsuoka–Nakai [5, 21] and is characterized through Eq. 25:

25 $$\begin{aligned} \frac{\left( \sigma _1+\sigma _2+\sigma _3\right) \left( \sigma _1\sigma _2+\sigma _1\sigma _3+\sigma _2\sigma _3\right) }{\sigma _1\sigma _2\sigma _3}=\frac{9-\sin ^2\varphi _{\mathrm{c}}}{1-\sin ^2\varphi _{\mathrm{c}}}. \end{aligned}$$

**Fig. 3:**
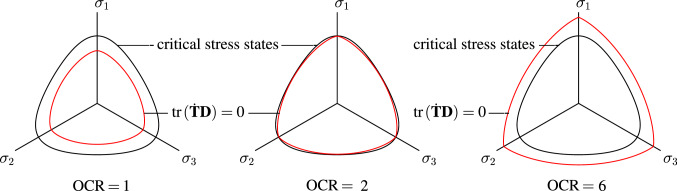
Surfaces of vanishing second-order work in the deviatoric plane. Obtained with barodesy [20] for various OCR-values. The critical state surface is also shown

In Fig. 3, vanishing second-order work is investigated with barodesy for Weald clay ($\varphi _{\mathrm{c}}=24^\circ$). The following results are obtained:

- For normally consolidated soil^4^ (OCR $=1$ and $e>e_{\text {c}}$) this cone lies inside the cone of critical stress states (Eq. 25).
- For $e=e_{\text {c}}$ ($p_{\text {e}}/p=2$ respectively), the cone of critical states and the cone of ${\hbox {tr}}{({\dot{{\mathbf {T}}}} {\mathbf {D}})}=0$ differ only slightly: However, the cone of vanishing second-order work lies inside the cone of critical stress states.
- For highly overconsolidated soil $e<e_{\mathrm{c}}$ (OCR = 6), the cone with ${\hbox {tr}}{({\dot{{\mathbf {T}}}} {\mathbf {D}})}=0$ lies outside the cone of critical stress states.

In Fig. 5a–c, stress locations in the deviatoric plane (${\hbox {tr}}{\mathbf {T}}=$ const), characterized by the Lode angle $\alpha _\sigma$ (Eq. 26), are further investigated. The Lode angle $\alpha _\sigma$ is defined as follows:

26 $$\begin{aligned}&\alpha _\sigma =\dfrac{1}{3}\arcsin {\dfrac{-3\sqrt{6}\det {{\mathbf {T}}^*}}{|{\mathbf {T}}^*|^3}} \nonumber \\&\quad {\hbox {with}}\quad {\mathbf {T}}^*={\mathbf {T}}-\frac{1}{3}\cdot {{\hbox {tr}}{\mathbf {T}}}\cdot {\mathbf {1}} \end{aligned}$$

$\alpha _\sigma =30^\circ$ holds for triaxial compression and $\alpha _\sigma =-30^\circ$ holds for triaxial extension. The angle $\beta$ between the directions of stress ${\mathbf {T}}^0$ and stretching ${\mathbf {D}}^0$ reads, cf. [7]:

27 $$\begin{aligned} \cos \beta ={\mathbf {T}}^0\cdot {\mathbf {D}}^0={\hbox {tr}}({\mathbf {T}}^0\,{\mathbf {D}}^0) \end{aligned}$$

For the undrained triaxial tests (with a prescribed ${\mathbf {D}}$, i.e. $D_1=-1$, $D_2=D_3=0.5$ and ${\hbox {tr}}{\mathbf {D}}^0=0$) in Fig. 1b $\beta$ is approximately $63.6^\circ$ and the mobilized friction angle is $\varphi _{W_2}\approx 21.3^\circ$, when $W_2=0$. A variation of ${\mathbf {D}}$ according to Eqs. 21–23 in order to find vanishing values of $W_2$ results in a slightly lower mobilized friction angle of $\varphi _{W_2}=21.1^\circ$. This variation was carried out at axisymmetric stress states. The critical friction angle of London clay is $22.6^\circ$.

For the non-conventional triaxial tests in Fig. 2, $\beta$ is approximately $86.4^\circ$ and the mobilized friction angle $\varphi _{W_2}$ is approximately $22.340^\circ$, which is also lower than the critical friction angle of Weald clay with $24^\circ$. A variation of ${\mathbf {D}}$ according to Eqs. 21–23 in order to find vanishing values of $W_2$ results in a slightly lower mobilized friction angle of $\varphi _{W_2}=22.336^\circ$.

**Fig. 4:**
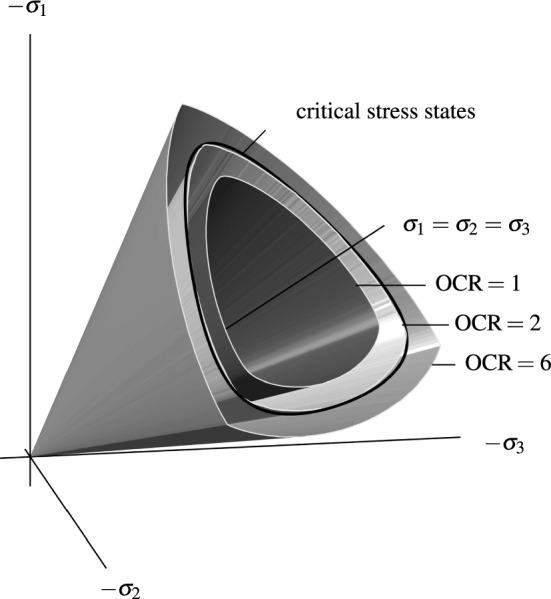
In barodesy, ${\hbox {tr}}{({\dot{{\mathbf {T}}}} {\mathbf {D}})}=0$ is described by a cone. A 3D representation of surfaces formed by ${\hbox {tr}}{({\dot{{\mathbf {T}}}} {\mathbf {D}})}=0$ for OCR $=1$, OCR $=2$ and OCR $=6$ according to Fig. 3 is shown. The critical stress surface of barodesy (Eq. 25) is added for ${\hbox {tr}}{\mathbf {T}}=-500$ kPa. In this plot, Weald clay is simulated with barodesy [20]

**Fig. 5:**
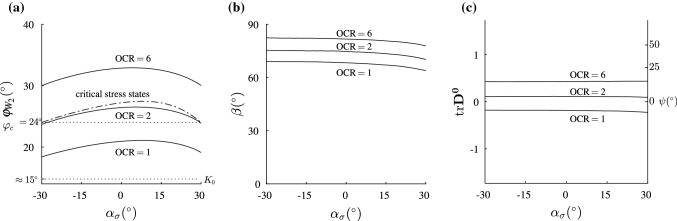
In **a**$\varphi _{W_2}$ according to Eq. 24 is shown for the different overconsolidation ratios, **b**$\beta$–$\alpha _\sigma$ plot, **c**${\hbox {tr}}{\mathbf {D}}^0$–$\alpha _\sigma$ plot. In this plot, Weald clay is simulated with barodesy [20]

Figures 4 and 5 give a more general view of the vanishing second-order work locus:

- In Fig. 4 a 3D representation of surfaces formed by ${\hbox {tr}}{({\dot{{\mathbf {T}}}} {\mathbf {D}})}=0$ for three different overconsolidation ratios (OCR $=1$, OCR $=2$ and OCR $=6$ according to Fig. 3) is shown. The cross section of the critical stress surface of barodesy (Eq. 25) with the deviatoric plane ${\hbox {tr}}{\mathbf {T}}=-500$ kPa is added.
- Figure 5a shows the mobilized friction angles $\varphi _{W_2}$ (obtained with $\sin \varphi _{\mathrm{m}}=\frac{T_{\text {min}}-T_{\text {max}}}{T_{\text {min}}+T_{\text {max}}}$) along the ${\hbox {tr}}({\dot{{\mathbf {T}}}}{\mathbf {D}})=0$ locus versus $\alpha _\sigma$. For normally consolidated samples (OCR $= 1$), the minimum mobilized friction angle is $\varphi _{W_2}\approx 18^\circ$, which is only $3^\circ$ higher than the mobilized friction angle under oedometric conditions estimated with Jáky’s relation.^5^ Similar results have been obtained with hypoplasticity [7]. For the OCR $=2$, the cone of vanishing second-order work lies slightly inside the cone of critical states, cf. Fig. 5a. For highly overconsolidated soil, $\varphi _{W_2}$ is higher than $\varphi _{W_2}$ of the critical stress surface, cf. Fig. 5a.
- Furthermore for OCR $=1$ the angle $\beta$ between normalized stress ${\mathbf {T}}^0$ and stretching ${\mathbf {D}}^0$ according to Eq. 27 is $63^\circ<\beta <69^\circ$, cf. Fig. 5b, the lower the void ratio (the higher the OCR), the higher the angle $\beta$. For highly overconsolidated soil, the angle $\beta$ ($77^\circ<\beta <82^\circ$) in Fig. 5b is higher than for slightly overconsolidated or normally consolidated soil.
- Figure 5c shows the dilatancy $\delta ={\hbox {tr}}{\mathbf {D}}^0$ in dependence of the Lode angle $\alpha _\sigma$. For normally consolidated clay (OCR $=1$), the behaviour is slightly contractant (${\hbox {tr}}{\mathbf {D}}^0\approx -0.2$). Note that ${\hbox {tr}}{\mathbf {D}}^0=0$ describes isochoric deformation and ${\hbox {tr}}{\mathbf {D}}^0=-1$ applies for oedometric compression. In addition, the angle of dilatancy $\psi$ is also shown in Fig. 5c.^6^ For an overconsolidation ratio of 2, clay is slightly dilatant (${\hbox {tr}}{\mathbf {D}}^0\approx 0.1$), for overconsolidated samples (OCR $=6$), ${\hbox {tr}}{\mathbf {D}}^0\approx 0.4$, cf. Fig. 5c. Arthur et al. [1] (cited in [33]) report that the angles of dilatancy $\psi$ in the shear plane in dense biaxial tests with sand were about $9^\circ \le \psi \le 30^\circ$. Simulations of overconsolidated samples (OCR $=2\ldots 6$) with barodesy result in angles of dilatancy in the range of $3^\circ<\psi <14^\circ$, see Fig. 5c. As in this article, clay samples with arbitrary overconsolidation ratios are investigated, only a qualitative comparison of the values for $\psi$ is possible.

Attention should be paid to normally consolidated/slightly overconsolidated soils, where second-order work may vanish inside the critical stress surface. The results provide a basis for finite element applications, as shown below.

## Finite element calculations

State of the art in geotechnical engineering are calculations of stress and strain fields with finite element approaches. Commercial finite element programs often allow assessment of stability by means of so-called *strength reduction analyses* ($\varphi$–*c* reduction). A frequently used approach to assess stability is to reduce the shear parameters until loss of convergence in the numeric calculation. This approach is ambiguous, as convergence depends not only on the stability but also on numerical issues as, e.g. incrementation. Investigations on the occurrence of ${\hbox {tr}}{({\dot{{\mathbf {T}}}} {\mathbf {D}})}\le 0$ in finite element calculations could give a more clear identification of instability [17, 22, 25, 27]. The here presented finite element calculations have been performed using Abaqus. For Abaqus, a user subroutine Umat for the material model barodesy is available [31]. As barodesy is not formulated in the framework of elasto-plasticity, a special strength reduction approach has been developed [32]. Second-order work has been made available as additional output variable in the user material subroutine. Thus, second-order work can be visualized easily in the abaqus framework for barodesy.

Note that in the finite element applications addressed here, $W_2$ is evaluated with the actual ${\dot{{\mathbf {T}}}}$ and ${\mathbf {D}}$ tensors. In other words, a search for the ${\mathbf {D}}$-tensor that minimizes $W_2$ has not been carried out, as it would render the calculations extremely lengthy. Consequently, the condition $W_2=0$ could be encountered even earlier. An analytical solution for the $W_2$ surfaces in stress space as developed by Niemunis [28] for hypoplasticity and applied by Meier et al. [22] is not yet archived for barodesy. However, the so obtained instability is still useful as indicator of failure.

### Biaxial tests

**Fig. 6:**
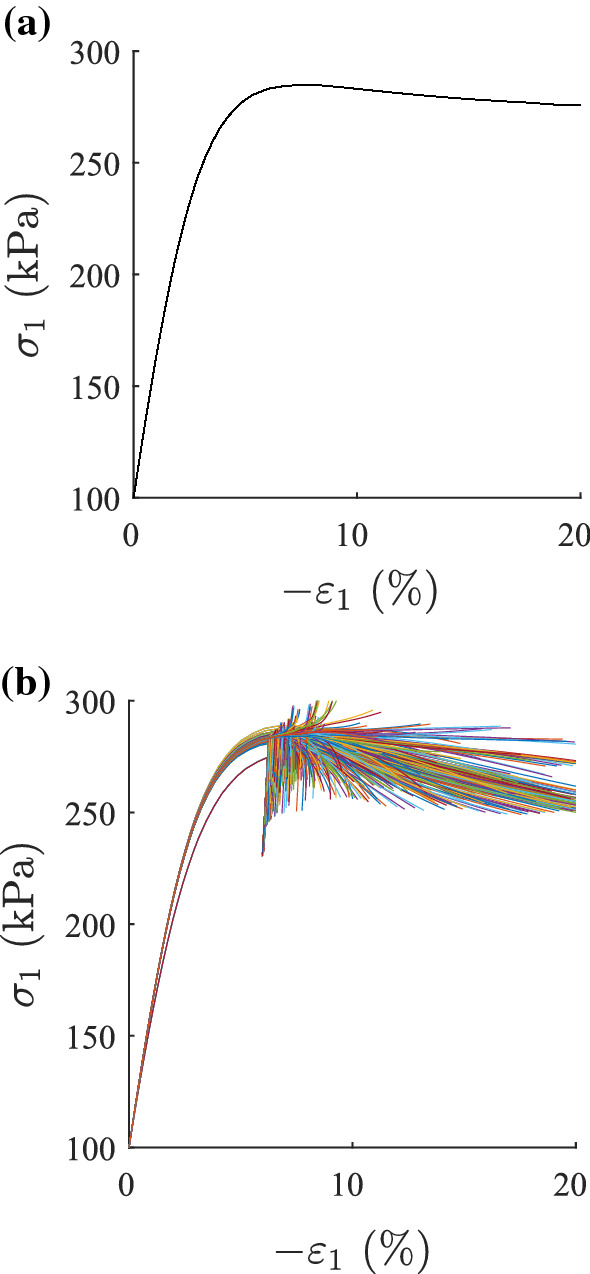
Axial stress $\sigma _1$–axial strain $\varepsilon _1$ relationship of all 200 elements of a biaxial test: in **a** the void ratio distribution is homogeneous, in **b** the void ratio distribution is inhomogeneous (one weak element)

**Fig. 7:**
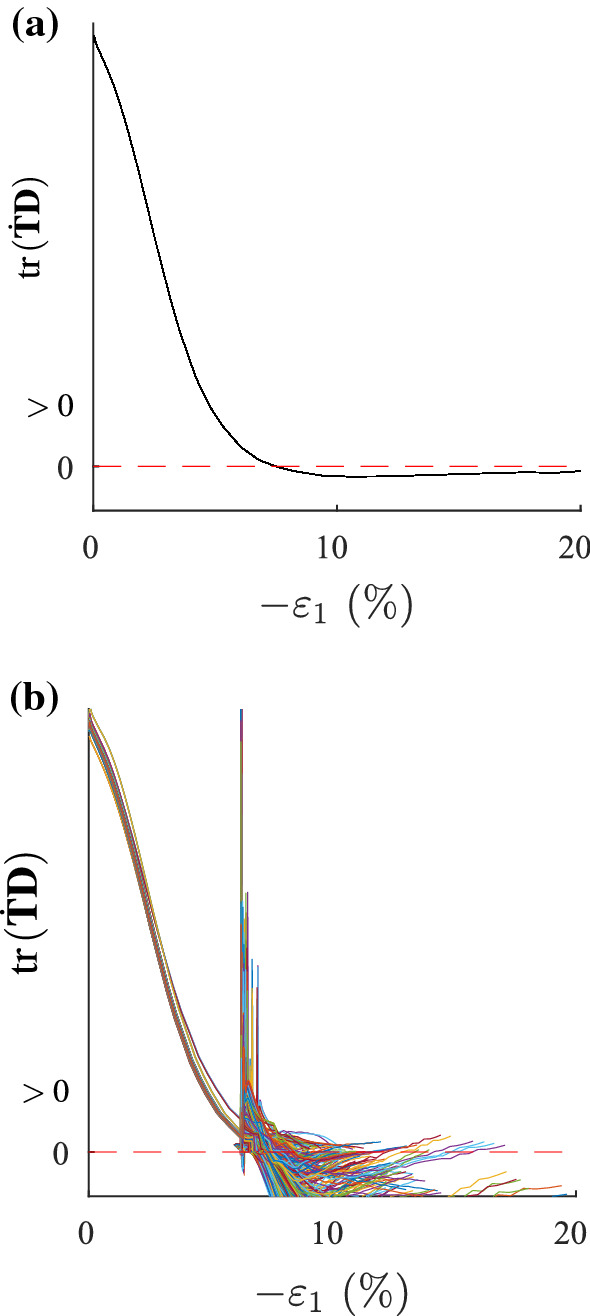
Second-order work evolution of all 200 elements of a biaxial test: in **a** the void ratio distribution is homogeneous, in **b** the void ratio distribution is inhomogeneous (one weak element)

The capability of modelling shear bands is an important property of material models. A first approach of visualizing shear bands in finite element calculations can be done on fine-meshed biaxial tests [14]. Finite element calculations of biaxial tests with barodesy have already been performed by Schneider-Muntau et al. [31], and the appearance of shear bands has been discussed. The same example is used in this article for shear band visualization with the second-order work criterion. For a biaxial test with a homogeneous void ratio distribution over 200 elements ($e_{{\text {ini}}} = 0.55$), all elements have the same deformation, see Fig. 6 for the stress–strain relationship and Fig. 7 for the second-order work distribution. Second-order work vanishes for all elements at the same calculation step, which corresponds to the peak at an axial strain of $\varepsilon _1=6.7\%$. Note that in laboratory tests inhomogeneous deformation exists from the very beginning of the test, be it small or pronounced.

**Fig. 8:**
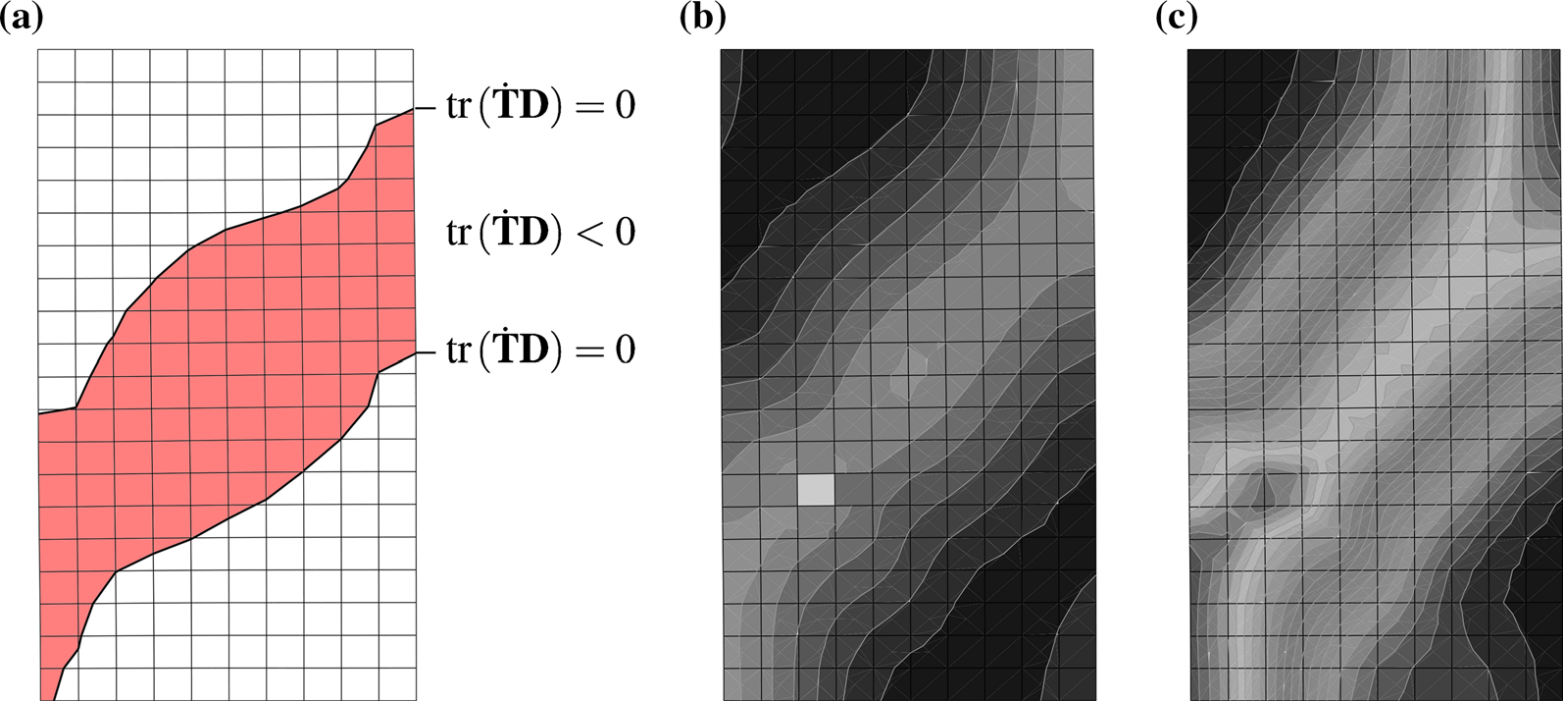
Biaxial test at a vertical strain $\varepsilon _1 = 6.7 \%$ (peak), shearband visualization: In **a** areas are marked where second-order work $\le 0$, **b** the void ratio distribution, in the range from 0.549 (black) to 0.561 (light grey), **c** the deviatoric strain, range from 0.07 (black) to 0.09 (light grey)

As to be expected, biaxial tests with an imperfection (a single looser element with $e_{{\text {ini}}} = 0.57$) show an inhomogeneous deformation from the very beginning of the test. In Fig. 6b, the stress–strain relationship of every single element is displayed. A similar behaviour for all elements is obtained until the peak. With continued deformation, the stress–strain relationships of each element get chaotic. At the peak, second-order work becomes negative for some elements. As can be seen in Fig. 8a, those elements form a band of vanishing/negative second-order work. Note that dead load controlled deformation would not be possible at this stage any more. The first occurrence of a continuous shearband is at 6.7% axial strain, which corresponds to the peak of the homogeneous void ratio distribution. At this stage, the shear band is not visible in the void ratio distribution, Fig. 8b, and can only be guessed in the distribution of deviatoric strains, see Fig. 8c. Visualizing shear bands in terms of deviatoric strain or void ratio distribution only indicate strain accumulation and are dependent on the visualization scale.

### Slope stability

**Fig. 9:**
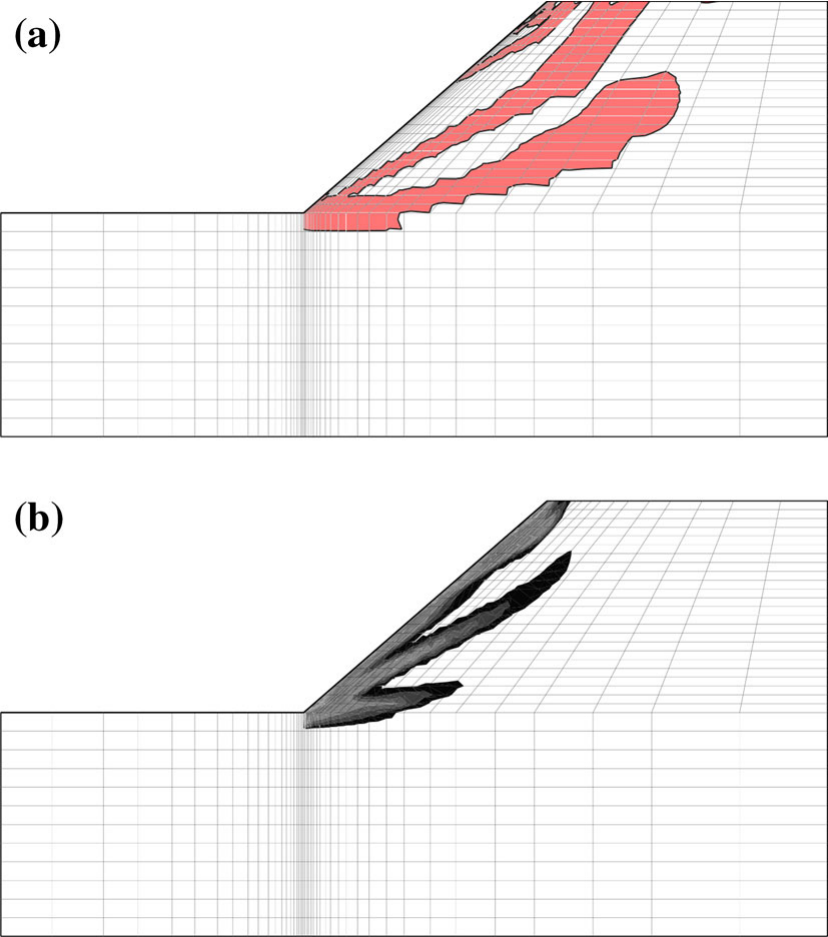
Slope at the moment of an appearance of a visible shear band by means of negative second-order work. In **a** areas are marked where second-order work $\le 0$**b** shows the void ratio distribution which ranges from 0.55 (black) to 0.99 (light grey), areas in white are below 0.55

Slope stability analyses in the framework of elasto-plasticity have been carried out, in order to identify unstable situations [4, 16, 27, 30]. Meier et al. [22] determine unstable areas of shallow slopes on the basis of a hypoplastic second-order work stability criterion according to Niemunis [28]. Slope stability finite element calculations with a strength reduction method for barodesy have been presented by Muntau et al. [32]. The material parameters $\varphi _{\text {c}}$ (critical friction angle) and *N* (ordinate intercept of the NCL) have been reduced gradually until the appearance of shear bands. The initial void ratio was set to 0.5 in all elements. Evaluating the shear band in terms of second-order work $\le 0$ yields the hatched areas in Fig. 9a. The band of vanishing/negative second-order work from bottom to top of the slope is clearly visible, contrary to the shear band in terms of void ratio distribution in Fig. 9b.

## Conclusions

Vanishing second-order work appears to be an suitable criterion for a situation where failure may occur. To evaluate ${\hbox {tr}}{(\dot{{\mathbf {T}}}}{\mathbf {D})}$, all possible ${\mathbf {D}}$-tensors and the pertinent ${\dot{{\mathbf {T}}}}$-tensors (resulting from a particular constitutive relation) should be investigated. This search is time-consuming and has been applied for the element tests in Figs. 3, 4 and 5: we varied the stretching tensor in the deviatoric plane in order to search for minimum values of second-order work. As soon as second-order work vanishes, the investigated stress state belongs to the searched boundary. For a constant overconsolidation ratio, vanishing second-order work is described by a cone. For normally consolidated to slightly overconsolidated soil, these cones lie inside the cone of critical stress states.

In barodesy, the second-order work approach showed—like in hypoplasticity and elasto-plasticity—that second-order may vanish at stress states inside the critical limit surface. It is obtained that for rather loose soils, second-order work vanishes inside the critical stress surface. Evaluating $W_2=0$ in the non-conventional triaxial tests in Fig. 2 with the actual ${\mathbf {D}}$-tensor and with a variation of ${\mathbf {D}}$, almost led to the same results for mobilized friction angles.

For finite element calculations, the variation of ${\mathbf {D}}$ is not feasible, so second-order work has been evaluated with the ${\mathbf {D}}$-tensor which is obtained from the equations of motion. For boundary value problems, an end-to-end band of vanishing second-order work marks the state where failure is imminent. In this article, second-order work has been investigated for the following boundary value problems:

1. Strain-controlled drained biaxial test with an initial imperfection (a slightly higher void ratio in one element). The stress–strain behaviour is similar for all elements until the peak, but then gets chaotic. At a certain strain—corresponding to the strain at the peak of a homogeneous sample—an area of vanishing or negative second-order work appears forming an end-to-end shear band.
2. Slope stability has been investigated by means of second-order work. The first appearance of an end-to-end band of $W_2\le 0$ can define system failure.

## Figures and Tables

**Fig. 10:**
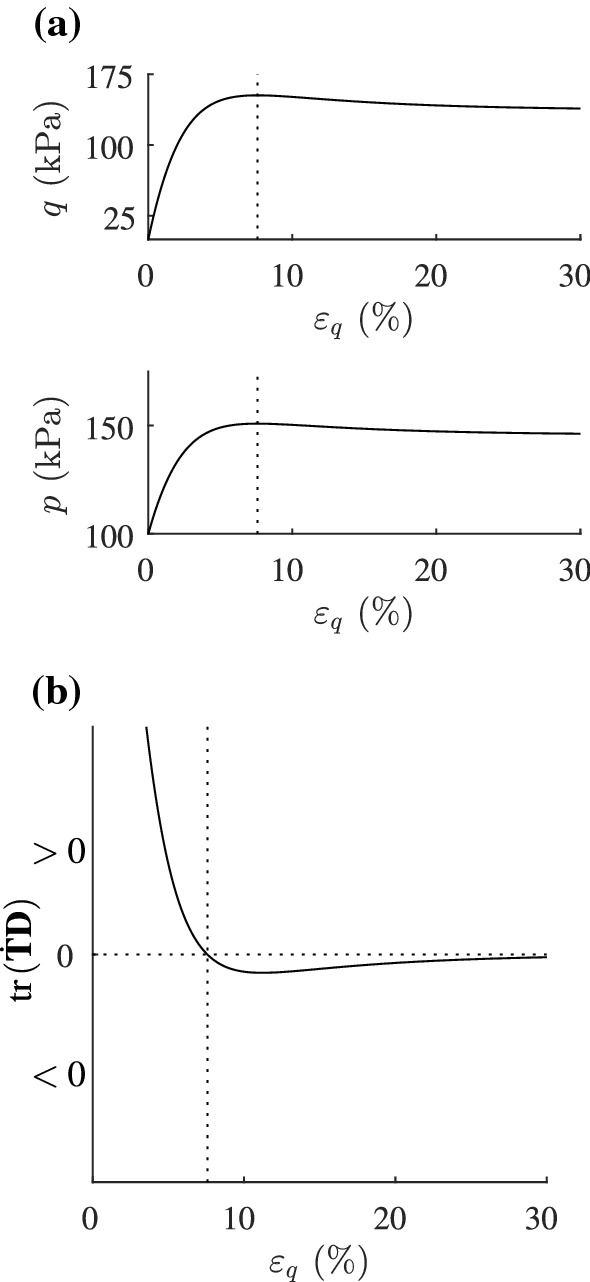
A drained triaxial test ($e_{{\text {ini}}}=0.55$ and $p_{{\text {ini}}}=100$ kPa) of Weald clay is simulated with barodesy. $W_2=0$ when ${\dot{q}}={\dot{p}} =0$. In **a** mean stress and deviatoric stress are plotted versus deviatoric strain, in **b** second-order work is plotted versus the deviatoric strain $\varepsilon _q$

## Appendix 1: Equations of barodesy

In this appendix, all equations of barodesy for clay [20] are summarized.

28 $$\begin{aligned} {\mathring{{\mathbf {T}}}}= h\cdot (f{\mathbf {R}}^0+g{\mathbf {T}}^0)\cdot |{\mathbf {D}}| \end{aligned}$$

29 $$\begin{aligned} {{\mathbf {R}}}= -\exp (\alpha {\mathbf {D}}^0) \quad {\hbox {with}}\quad \alpha =\dfrac{\ln K}{\sqrt{3/2-{{\hbox {tr}}{\mathbf {D}}^0}^2/2}}\end{aligned}$$

30 $$\begin{aligned} K= 1-\dfrac{1}{1+c_1(m-c_2)^2} \quad {\hbox {with}}\quad m=\dfrac{-3{\hbox {tr}}{\mathbf {D}}^0}{\sqrt{6-2{{\hbox {tr}}{\mathbf {D}}^0}^2}}\end{aligned}$$

31 $$\begin{aligned} h= c_3|{\mathbf {T}}|^{c_4}\end{aligned}$$

32 $$\begin{aligned} f= c_6\cdot \beta \cdot {\hbox {tr}}{\mathbf {D}}^0-\frac{1}{2} \end{aligned}$$

33 $$\begin{aligned} g= (1-c_6)\cdot \beta \cdot {\hbox {tr}}{\mathbf {D}}^0+\left( \frac{1+e}{1+e_{\mathrm{c}}}\right) ^{c_5}-\frac{1}{2}\end{aligned}$$

34 $$\begin{aligned} e_{\mathrm{c}}= \exp \left( N-\lambda ^*\ln \frac{-2/3 \quad {\hbox {tr}}{\mathbf {T}}}{\sigma ^*}\right) -1\end{aligned}$$

35 $$\begin{aligned} \beta= -\frac{1}{c_3\varLambda }+ \frac{1}{\sqrt{3}}2^{c_5\lambda ^*}- \frac{1}{\sqrt{3}} \end{aligned}$$

36 $$\begin{aligned} \varLambda= -\frac{\lambda ^*-\kappa ^*}{2\sqrt{3}}{\hbox {tr}}{\mathbf {D}}^0+\frac{\lambda ^*+\kappa ^*}{2} \end{aligned}$$

The constants $c_1$–$c_6$ can be determined on the basis of the critical state soil mechanics parameters $\varphi _{\mathrm{c}}$, *N*, $\lambda ^*$ and $\kappa ^*$, cf. [20].

## Appendix 2: Drained triaxial test

For axisymmetric loading conditions, Eq. 2 can be rewritten as:

37 $$\begin{aligned} {\hbox {tr}}({\dot{{\mathbf {T}}}}{\mathbf {D}})={\dot{T}}_{1}\cdot {\dot{\varepsilon }}_1+2\cdot {\dot{T}}_{2}\cdot {\dot{\varepsilon }}_2= -{\dot{p}} \cdot {\dot{\varepsilon }}_{\text {vol}}-{\dot{q}}\cdot {\dot{\varepsilon }}_q \end{aligned}$$

In the peak state of a drained triaxial test, we have ${\dot{p}}={\dot{q}}=0$. Thus, at the peak ($\varphi _{\mathrm{m}}=25.65^\circ$) of a drained triaxial test, second-order work vanishes, cf. Fig. 10. A variation of ${\mathbf {D}}$ according to Eqs. 21–23 in order to find vanishing values of $W_2$ results in a slightly lower mobilized friction angle of $\varphi _{W_2}=25.57^\circ$. The critical friction angle of Weald clay is $24^\circ$. It is interesting to investigate whether the condition ${\hbox {tr}}({\dot{{\mathbf {T}}}}{\mathbf {D}})=0$ can be encountered even *before* the peak. This is in fact the case with conventional triaxial tests [11] and can be experimentally shown with so-called non-conventional drained triaxial tests, cf. Sect. 4.2.

## Footnotes

1 Hypoplasticity and barodesy are constitutive models which do not use the standard notions of elasto-plastic models (such as elastic region, yield function, flow function, ...). Instead, the effective co-rotated stress rate ${\mathring{{\mathbf {T}}}}$ is given as a function of stretching ${\mathbf {D}}$ and of effective stress ${\mathbf {T}}$, optionally complemented by further variables, as the void ratio *e*.

2 In this article, the simulations are carried out with barodesy for clay. All equations and a detailed description can be found in the open access article by Medicus and Fellin [20].

3 In this article, the mobilized friction angle when second-order work vanishes is denoted as $\varphi _{W_2}$.

4 It is common to define the overconsolidation ratio (OCR = $p_{\text {e}}/p$) by means of the so-called *Hvorslev’s equivalent consolidation pressure*$p_{\text {e}}=\exp \left( \frac{N-\ln (1+e)}{\lambda ^*}\right)$, divided by the actual mean stress *p*. $p_{\text {e}}$ is the value of mean stress on the isotropic normal consolidation line which refers to the current specific volume $(1+e)$. If $p/p_{\text {e}} =$  const in a deviatoric plane ($p=$  const), then the void ratio *e* is constant.

5 From the earth pressure coefficient at rest $K_0=1-\sin \varphi _{\mathrm{c}}=\dfrac{1-\sin \varphi _{\mathrm{m}}}{1+\sin \varphi _{\mathrm{m}}}$ we obtain with $\varphi _{\mathrm{c}}=24^\circ$ the mobilized friction angle under oedometric conditions $\varphi _{W_2}=\arcsin \dfrac{\sin \varphi _{\mathrm{c}}}{2-\sin \varphi _{\mathrm{c}}}\approx 15^\circ$.

6 Note that the angle of dilatancy $\psi$ in Fig. 5c strictly applies for axisymmetric compression (i.e. $\alpha _\sigma =30^\circ$) only. Experimental studies by Nakai [24] showed that $0<\alpha _\sigma <15^\circ$ for plane strain conditions. These findings correspond to results obtained with barodesy [21].
